# A Smart Realtime Service to Broadcast the Precise Orbits of GPS Satellite and Its Performance on Precise Point Positioning

**DOI:** 10.3390/s20113276

**Published:** 2020-06-08

**Authors:** Dehai Li, Wei Yan, Jinzhong Mi, Yamin Dang, Yunbin Yuan, Xingli Gan

**Affiliations:** 1 Chinese Academy of Surveying and Mapping, No.28, Lianhuachi West Road, Beijing 100830, China; lidh@casm.ac.cn (D.L.); Dangym@casm.ac.cn (Y.D.); 2 Tianjin Institute of Surveying and Mapping, Changling Road, Tianjin 300381, China; yanweigps@gmail.com; 3 Innovation Academy for Precision Measurement Science and Technology, Chinese Academy of Science, Xiaohongshan West Road, Wuhan 430071, China; yybgps@asch.whigg.ac.cn; 4 The 54th Research Institute of China Electronics Technology Group Corporation, Shijiazhuang 050081, China; ganxingli@163.com

**Keywords:** GPS, RTCM, real-time services, precise orbits transmission, real-time precise point positioning

## Abstract

At present, Global Position System (GPS) navigation ephemeris mainly broadcasts satellite orbits with meter-level precision for standard point positioning and precise relative positioning. With the rapid development of real-time precise point positioning (PPP), the receiver or smartphone has begun to demand more and more convenient, continuous, and reliable access to real-time services of precise orbits. Therefore, this study proposes a solution of utilizing the 18-parameter ephemeris to directly broadcast ultra-rapid precise predicted orbits with centimeter-level precision for real-time PPP. For the first time in GPS, the difference in the PPP results between the precise orbits and the calculated orbits broadcasted from the generated ephemeris parameters is supplied as follows: (1) During the validity period of 2 h, root mean square (RMS) of the relative distance offsets between the results of PPP with the precise orbits and the results of PPP the 18-parameter ephemeris is only 0.0098 m. (2) Within 15 min after the validity period of 2 h, RMS of the relative distance offsets between the results of PPP with the precise orbits and the results of PPP with the predicted orbits by 18-parameter ephemeris is only 0.0057 m. Consequently, the 18-parameter ephemeris is feasible and advisable to broadcast precise predicted orbits for real-time PPP applications. Compared with the classic precise orbits broadcast mode with the orbit corrections defined by the radio technical commission for maritime services standards 10403.2 (RTCM), the mode of broadcasting the precise orbits with the 18-parameter ephemeris achieved the following improvements in convenience, continuity, and reliability: (1) The calculation of satellite position is the same as that of the navigation ephemeris excluding the additional correction operations required to the RTCM; (2) the amount of broadcast parameters was reduced by 20 times; (3) the length of the validity period was expanded 120 times, where the longer valid period helped to overcome the orbit corrections loss caused by RTCM stream failures; and (4) within 15 min after the validity period, the predicted orbits with an accuracy of 2 cm could still be provided by the 18-parameter ephemeris, which can ensure the real-time services of precise orbits in the case of a 15 min communication interruption of the RTCM orbit correction data stream.

## 1. Introduction

In recent years, precision point positioning (PPP) technology has been widely studied, and the products of PPP are applied in the various domains of timing, geodesy, geophysics, seismology, atmology, and so on. By using precise orbits and clocks in the posterior mode, PPP can provide accurate station coordinates, clock errors, tropospheric delay, and ionospheric delay. PPP is becoming more and more complete, and the main interest has turned to real-time PPP [1,2,3,4].

A receiver or smartphone can be utilized to implement real-time PPP with precise predicted orbits and real-time estimated clocks to achieve centimeter-level real-time positioning services [5]. In this way, the precise orbits and clocks must be broadcast to the receiver in real-time. Thereby, the real-time transmission of precise orbits and clocks is a necessary step to guarantee the real-time PPP. In particular, the lightweight performance on the precise orbits broadcast will directly affect the transmission load and the acquisition delay. Moreover, for real-time services of precise orbits, the continuity and reliability will be the determining factors on the actual performance of real-time PPP. At present, the real-time services of precise orbits and clocks are implemented with the data stream defined by the radio technical commission for maritime services standards 104,03.2 (RTCM) [6].

On one hand, RTCM provides a real-time mode of broadcasting precise clocks, and the broadcast message is the clock corrections. The clock corrections are the differences between the clock errors of navigation ephemeris from the navigation messages and the precise clock errors. Due to the fast change of a satellite clock, the characteristic of clock corrections is different from that of orbit corrections. Consequently, clock corrections are difficult to predict with a precision of sub-nanosecond level [7,8,9,10]. Therefore, the clock corrections are estimated in real-time and broadcast at high frequency to support real-time precise positioning services [11].

On the other hand, RTCM provides a real-time mode of broadcasting precise orbits, and the broadcast message is the orbit corrections and its change rate. The orbit corrections are the differences between the calculated orbits from the navigation ephemeris and the precise orbits. The navigation ephemeris is decoded from the navigation messages, and the orbit accuracy provided by the GPS navigation ephemeris in the navigation messages is about 1 m [12,13]. The International GNSS Service (IGS) provides ultra-rapid predicted orbits with an accuracy of 5 cm. The ultra-rapid orbits are updated every 6 h in advance [14,15]. The ultra-rapid orbits can be used as a real-time precise orbits for real-time PPP [16]. The orbit corrections are generated from the navigation ephemeris and the ultra-rapid orbits. The correction rate is generally treated as 0 by default. Then, the orbit corrections are transmitted according to the RTCM with the update period of 60 s [17]. Moreover, IGS has established the real-time services (RTS) to provide real-time data streams of orbit and clock corrections, which substantially improve the availability and precision of real-time PPP. Fortunately, with the help of the Multi-GNSS Experiment (MGEX), IGS and Bundesamt Kartographieund Geodesie (BKG) are continuing to upgrade the real-time services of orbits and clocks to augment real-time PPP.

As an alternative but improved approach, the 18-parameter ephemeris may be beneficial to enhance the performance of broadcasting the precise orbits relative to the RTCM to serve real-time PPP. In order to broadcast the navigation ephemeris of geosynchronous Earth orbit (GEO), Du et al. [18] investigated the performance of 18-parameter ephemeris, and solved the singular problem caused by the GEO inclination angle close to 0. Those works demonstrate the promising potential of the 18-parameter ephemeris. In contrast to the existing research of navigation ephemeris application, this study proposes a GPS service mode in which the generated 18-parameter ephemeris is directly applied to real-time PPP for the receiver and smartphone, and quantitatively investigates the difference in the PPP results produced by this service mode of broadcasting precise orbits.

There are many differences in terms of the implemented background and the applied object. The traditional GPS navigation ephemeris is mainly used to broadcast satellite orbits with a precision of about 1 m, and serve standard point positioning with meter-level precision and precise relative positioning. Specifically, the accuracy of navigation ephemeris is variant from different GNSS [19]. With the progress of GNSS construction, Galileo has brought more accurate navigation ephemeris, whose accuracy of broadcast orbits is almost three times relative to that of GPS. Galileo’s advancement in navigation ephemeris provides inspiring support to the real-time PPP services. Using the broadcast orbits and clocks from Galileo navigation ephemeris, real-time PPP can reach 7 cm in horizontal and 10 cm in vertical accuracy. This result exhibits a promising reference for GPS [20]. On this basis, a new GPS service is proposed to generate the ephemeris to broadcast ultra-rapid precise orbits with a precision of about 5 cm, and is supplied for the centimeter-level real-time PPP on the receiver and smartphone. For the first time in GPS, this paper provides the difference in the PPP results between the precise orbits and the calculated orbits with the generated ephemeris parameters, and the deviations resulted from the mode of broadcasting precise orbits with the generated ephemeris were carefully evaluated and analyzed in terms of orbit errors and PPP offsets. Consequently, some helpful findings are supplied to real-time PPP.

Unlike the classic mode provided by RTCM to broadcast precise orbits, this study suggests a smart mode of directly broadcasting precise orbits with the generated ephemeris parameters, which reduces the number of broadcast parameters and transmission burden, and improves the convenience, continuity, and reliability of real-time service of precise orbits. The classic RTCM mode broadcasts the corrections and its rate of the precise orbits with respect to the navigation ephemeris. Unfortunately, when the receiver used the Internet to receive the orbit correction data streams, continuous success is hardly guaranteed, since it is difficult to ensure that the transmission network is always normal [10]. Occasionally, when the network link or data stream is suspended and reconnected, the missing orbit correction data during the abnormal period brings about the interruption and discontinuity of real-time services of precise orbits. In addition, the validity period of the RTCM orbit corrections is as short as 60 s. Accuracy of the predicted orbits after the validity period are hardly guaranteed with the previous correction data. If the correction data stream is suspended for more than 5 min, precise orbit services become unavailable [21].

Fortunately, the aforementioned disadvantages would be mitigated by the smart mode of broadcasting precise orbits with the 18-parameter ephemeris. By taking the advantages of broadcast ephemeris to realize precise orbits transmission in real-time services, the contributions are resulted in improving the performances of lightweight, convenience, continuity, and reliability. In fact, all receivers and smartphones already employ the navigation ephemeris from the GPS signal, which provide the functions of receiving, decoding, and calculating navigation ephemeris. If the real-time precise orbits are broadcast by the same format of navigation ephemeris, the receiver can use the precise ephemeris to carry out the real-time precise positioning application without additional changes [22]. The broadcast ephemeris requires only 18 parameters within the 2 h validity period, which greatly reduces the number of RTCM orbit correction parameters during the same period. In addition, a set of broadcast ephemeris can cover precise orbits within the validity period of 2 h at a time, which provides continuous and accurate satellite positions, and helps to avoid the problem of the interruption of precise orbit services caused by orbit correction data stream failures or absences.

The rest of this paper is organized as follows. First, a method of generating broadcast ephemeris parameters is formed. By explaining the content of the 18-parameter ephemeris, the observation model and estimation model are established to generate the broadcast ephemeris parameters. Second, the performance of the 18-parameter ephemeris broadcasting precise orbits and its improvement over the traditional 16-parameter ephemeris are studied. The ephemeris parameters are generated from the GPS precise orbits. With the generated ephemeris, the satellite orbits are calculated to obtain the offsets relative to the original precise orbits. By investigating the statistical results of orbit offsets, the tests and analysis are conducted in terms of the fitting accuracy during the validity period, the predicted accuracy after the validity period, and the stability of ephemeris parameters generation. Finally, PPP tests are carried out with the generated 18-parameter ephemeris. The results of PPP with the calculated orbits from the 18-parameter ephemeris during the validity period are compared with the results of PPP with the precise orbits. Meantime, the results of PPP with the predicted orbits from the 18-parameter ephemeris after the validity period are compared with the results of PPP with the precise orbits. Then, the feasibility of the 18-parameter ephemeris broadcasting precise orbits for real-time PPP is verified.

It should be mentioned that the 18-parameter ephemeris and 16-parameter ephemeris are suitable for medium earth orbit (MEO) satellites. All GPS satellites use MEO. In this paper, GPS precise orbits and observation data were adopted by the tests of generating ephemeris parameters to broadcast precise orbits and the PPP tests with the generated ephemeris. The numerical results are only applicable to GPS, while the relevant testing conclusions can also be taken as a reference for other GNSS. Due to the space limitation, the broadcast of real-time precise orbits from others including GEO and inclined geosynchronous orbit (IGSO) can be planned and conducted in the future.

## 2. Method

At present, broadcast ephemeris mainly includes 16-parameter ephemeris and 18-parameter ephemeris. The 18-parameter ephemeris adds two parameters relative to the 16-parameter ephemeris. One is the change rate in the semi-major axis, and the other is the rate of mean motion difference from the computed value. In this section, first, the composition and definition of ephemeris parameters are explained according to the interface specifications of GPS [23]. Second, the observation model of ephemeris parameters is established by taking the precise orbits as the observations. Finally, the estimation model is built by setting the ephemeris parameters as the estimated parameters. Then, a method of generating ephemeris parameters is formed.

### 2.1. Overview of Ephemeris

Early GPS satellites adopted 16-parameter ephemeris, and all receivers supported this ephemeris [24]. Afterward, a refined 18-parameter ephemeris was defined to improve the performance of GPS navigation ephemeris. The content of 18-parameter ephemeris includes the mean anomaly at reference time $M_{0}$; mean motion difference from computed value $\Delta n_{0}^{}$; eccentricity *e*; semi-major axis difference at reference time ΔA with respect to $A_{ref}$ = 26,559,710 m; rate of mean motion difference from computed value $\Delta \dot{n}$; change rate in semi-major axis $\dot{A}$; longitude of ascending node of orbit plane at weekly epoch $\Omega_{0}$; argument of perigee *w*; inclination angle at reference time *i*; rate of right ascension difference $\Delta \dot{\Omega }$ with respect to $\Delta {\dot{\Omega }}_{ref}=-2.6\times 10^{-9}$ semi-rad/s; rate of inclination angle $\dot{i}$; amplitudes of the cosine and sine harmonic correction terms to the argument of latitude $c_{uc}$ and $c_{us}$; amplitudes of the cosine and sine harmonic correction terms to the orbit radius $c_{rc}$ and $c_{rs}$; amplitudes of the cosine and sine harmonic correction terms to the angle of inclination $c_{ic}$ and $c_{is}$; and ephemeris reference time $t_{oe}^{}$. The 16-parameter ephemeris does not contain the rate of mean motion difference from the computed value $\Delta \dot{n}$ and change rate in semi-major axis $\dot{A}$, where the semi-major axis difference at reference time ΔA turns to the square root of the semi-major axis $\sqrt{A}$, and the rate of right ascension difference $\Delta \dot{\Omega }$ turns to the rate of right ascension $\dot{\Omega }$.

In the calculation model of 18-parameter ephemeris, some new algorithms are applied, which are shown as follows:

(1) $$t_{k}^{}=t-t_{oe}^{}$$

(2) $$A_{0}^{}=A_{ref}^{}+\Delta A$$

(3) $$A_{k}^{}=A_{0}^{}+\dot{A}t_{k}^{}$$

(4) $$\Delta n_{k}^{}=\Delta n_{0}^{}+0.5\Delta {\dot{n}}_{0}^{}t_{k}^{}$$

(5) $$\dot{\Omega }={\dot{\Omega }}_{ref}^{}+\Delta \dot{\Omega }$$

(6) $$\Omega_{k}^{}=\Omega_{0}^{}+(\dot{\Omega }-{\dot{\Omega }}_{e}^{})t_{k}^{}-{\dot{\Omega }}_{e}^{}t_{oe}^{}$$

where *t* is the calculation epoch; $A_{k}^{}$ is the semi-major axis on the calculation epoch; $\Delta n_{k}^{}$ is the correction of mean motion; $\Omega_{k}^{}$ is the corrected longitude of ascending mode on the calculation epoch; and ${\dot{\Omega }}_{e}^{}$ is the Earth’s rotation rate.

### 2.2. The Observation Model of Ephemeris Parameters

By linearizing the calculation model of ephemeris [25,26], the observation model for the ephemeris parameters can be established. The satellite positions provided from the precise orbits are treated as the observation values, and the corrections of ephemeris parameters are set as the unknown parameters to be estimated. Thereby, the observation model can be established to estimate the ephemeris parameters. However, in the ephemeris parameters estimation, the ephemeris parameter of reference time is set as the known value and does not need to be estimated, so the parameters to be estimated in the generation of the 18-parameter ephemeris include only 17 parameters. Then, the observation model of 18-parameter ephemeris is expressed as:

(7) $$\begin{array}{c} R_{i}(t)=R_{{}_{i}}^{0}(t)+\Delta R_{i}(t)\\ =R_{{}_{i}}^{0}(t)+\sum_{j=1}^{17}dR_{ij}(t)d_{j}\\ =R_{{}_{i}}^{0}(t)+\frac{\partial R_{i}}{\partial \Delta A}d\Delta A+\frac{\partial R_{i}}{\partial \dot{A}}d\dot{A}+\frac{\partial R_{i}}{\partial e}de+\frac{\partial R_{i}}{\partial i_{0}}di_{0}+\frac{\partial R_{i}}{\partial \Omega_{0}}d\Omega_{0}+\frac{\partial R_{i}}{\partial w}dw+\frac{\partial R_{i}}{\partial M_{0}}dM_{0}+\frac{\partial R_{i}}{\partial \Delta n_{0}}d\Delta n_{0}\\ +\frac{\partial R_{i}}{\partial \Delta {\dot{n}}_{0}}d\Delta {\dot{n}}_{0}+\frac{\partial R_{i}}{\partial \Delta \dot{\Omega }}d\Delta \dot{\Omega }+\frac{\partial R_{i}}{\partial \dot{i}}d\dot{i}+\frac{\partial R_{i}}{\partial c_{us}}dc_{us}+\frac{\partial R_{i}}{\partial c_{uc}}dc_{uc}+\frac{\partial R_{i}}{\partial c_{is}}dc_{is}+\frac{\partial R_{i}}{\partial c_{ic}}dc_{ic}+\frac{\partial R_{i}}{\partial c_{rs}}dc_{rs}+\frac{\partial R_{i}}{\partial c_{rc}}dc_{rc} \end{array}$$

where $R_{i}(t)$ (*i* = 1, 2, 3) is the coordinate component of satellite position in earth-centered, earth-fixed coordinate (ECEF) on the epoch *t*; $R_{i}^{0}(t)$ is the initial value of satellite position calculated with the initial ephemeris; $\Delta R_{i}(t)$ is the influence of all ephemeris parameter corrections on satellite coordinate; $dR_{ij}(t)$ is the partial derivatives of $R_{i}(t)$ with respect to ephemeris parameters; $d_{j}$ is the ephemeris parameter correction; $d\dot{A}$ is the correction of $\dot{A}$, and it is same for others; and $t_{oe}^{}$ is deployed as the referenced epoch for the generation of ephemeris parameters without correction. The partial derivatives of the satellite coordinate component $R_{i}(t)$ with respect to the corresponding ephemeris parameters are listed in the Appendix A.

### 2.3. The Estimation Model for Ephemeris Parameters

Based on the aforementioned observation model, the error equation of 18-parameter ephemeris is constructed as:

(8) V=BX−L

where *V* is the vector of the observation noises of satellite positions from the precise orbits. *B* is the design matrix. The matrix elements are obtained by the partial derivatives of the satellite position with respect to the ephemeris parameters as Equation (7). *L* is the vector of the differences between the observed values and the approximately calculated values. The observed values are the satellite positions provided by the precise orbits, and the calculated values are the satellite positions calculated by the initial ephemeris parameters. *X* is the vector of the ephemeris parameter corrections.

With initial values of ephemeris parameters, the former error equation is solved by iterative least-squares method, and the optimal estimates of ephemeris parameters are obtained. Then, the precise orbits are transmitted by the way of broadcasting 18-parameter ephemeris, and the needs of the real-time services of precise orbits can be fulfilled to real-time PPP.

As well as the central gravity, GPS satellite orbits are also manipulated by many perturbations such as non-spherical perturbation, planetary gravity, solar radiation pressure, atmospheric drag, earth radiation pressure, and so on. These perturbation forces change continuously with the satellite positions, and produce significant long-term, long-period, and short-period effects on the orbits. As such, all perturbation effects are treated as the short-period term to be modeled and represented with the 18-parameter ephemeris [18]. The 18-parameter ephemeris adds the rate of the semi-major axis and the rate of mean motion, which are more suitable for describing the perturbations of GPS satellite orbit parameters. In fact, since the GPS satellites are in deep resonance with the Earth’s rotation, this resonance causes a periodic variation of very long repeatability on the semi-major axis, which is treated as a small drift in the 2 h period to be modeled by the rate of the semi-major axis [25,26]. 

With the increase in arc length, the ability of the ephemeris model to represent longer arcs will decline, and the fitting accuracy will decrease rapidly [24]. Therefore, by setting a shorter validity period such as 2 h, the accuracy of the orbits fitting with the ephemeris is ensured during the validity period. In the ephemeris parameter generation, since the reference time of the ephemeris parameters is set to a known time and does not need to be estimated, the estimated parameters only include 17 parameters. The observations are the three-dimensional coordinates sequence of the satellite in ECEF, and an observation from a single epoch provides three observation equations. Thereby, at least six epochs of satellite positions are required to estimate the ephemeris parameters. For precise orbits of 15 min interval provided by IGS, orbits with a length of 90 min would fulfill the minimum requirements of the 18-parameter ephemeris generation.

## 3. Test Description

In order to evaluate the accuracy of the generated ephemeris broadcasting precise orbits and the influence on the precision of PPP with the generated ephemeris [19], simulation tests were designed in this section. The experimental results were obtained from real GPS observation data and the actual precise orbits used to simulate the real-time processing. The actual precise orbits from IGS products were processed as the generated ephemeris parameters. According to the real precise orbits, a comparison test was arranged to assess the fitting and predicting accuracies of the generated ephemeris. Furthermore, using the generated ephemeris to provide the real-time services of precise orbits, PPP experiments were carried out. The real GPS observation data were tested by the PPP with the generated ephemeris, and the precision of PPP with the calculated orbits by the generated ephemeris is demonstrated. The differences in the PPP results between the generated 18-parameter ephemeris, the generated 16-parameter ephemeris, and the precise orbits were compared, and the deviations that resulted from the generated ephemeris broadcasting the precise orbits were supplied. In practical application, the ultra-rapid precise predicted orbits provided by IGS can be used in generating ephemeris parameters to broadcast the precise orbits and achieve real-time services of PPP.

**Figure 1:**
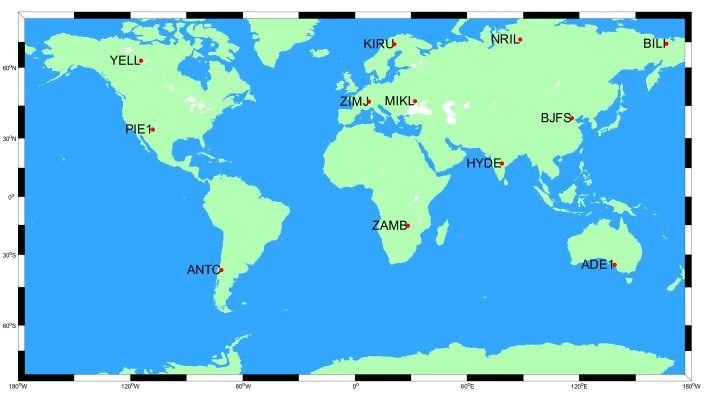
The distribution of 12 IGS stations in the PPP tests.

In the tests, the 16-parameter ephemeris and 18-parameter ephemeris were adopted to broadcast precise orbits, respectively. The valid periods of ephemeris were set to 2 h and 3 h. Specifically, the GPS constellations and arc segments covered as many as possible. Precise orbits of all GPS satellites over two days provided by IGS on 5–6 July 2017 were employed to generate the ephemeris parameters for broadcasting. In the PPP tests, the dual-frequency data were collected from 12 IGS stations globally distributed, as shown in Figure 1. Particularly, PPP tests utilize conventional ionospheric combinations, precise clock products, and orbits including the precise orbits, the calculated orbits from the generated ephemeris within the validity period, and the predicted orbits of the generated ephemeris after the validity period, respectively.

The simulation tests were designed to assess the accuracy of the generated ephemeris broadcasting precise orbits. The testing periods covered the inner and outer periods of the ephemeris validity. On one hand, the tests during the validity period were designed to assess the accuracy of ephemeris fitting precise orbits, the stability of generating ephemeris parameters from precise orbits, and the influence of different validity periods on fitting accuracy. On the other hand, the tests exceeding the validity period were planned to assess the prediction accuracy of the ephemeris, the prediction stability, and the influence of different validity periods on the prediction accuracy. The radial, along-track, and cross-track offsets (RAC) were adopted to evaluate the differences between the precise orbits and the calculated orbits by the generated ephemeris.

The PPP tests were designed to assess the performance of the generated ephemeris on precise point positioning. During the ephemeris validity period, the PPP tests were employed to assess the offsets between the results of PPP with the precise orbits and that of the calculated orbits from the generated ephemeris, and the positioning errors deduced from the results of PPP with the calculated orbits relative to the known position. In the same way, over the validity period, PPP tests were arranged to assess the offsets between the results of PPP with the precise orbits and that of the predicted orbits from the generated ephemeris, and the positioning errors. The accuracy and convergence of PPP depend on many factors such as the accuracies of orbit and clock, and the quality of the observation data. This study mainly focused on the difference between PPP with the generated ephemeris and PPP with the precise orbits, and the offsets because of the generated ephemeris replacing the precise orbits. Therefore, the impact of the generated ephemeris broadcasting precise orbits on PPP performance was carefully validated in this paper.

## 4. Experimental Results and Analysis

### 4.1. Results of the Generated Ephemeris Fitting Precise Orbits

The fitting performances of 18-parameter ephemeris and 16-parameter ephemeris were tested by precise orbits from all GPS satellites and different arcs. The ephemeris parameters can be generated from the precise orbits. Since the ultra-rapid predicted orbits are updated with the period of six hours, the validity period of the generated ephemeris should be an integer divisible by six. In the tests, the validity period of broadcast ephemeris was set for 2 and 3 h. From the precise orbits released by IGS, the GPS satellite positions were treated as the observation values, and the generation method was utilized to obtain the broadcast ephemeris parameters. During the validity period over two days, the satellite positions were calculated with the 18-parameter ephemeris and 16-parameter ephemeris. The offsets were obtained between the calculated orbits and the precise orbits. The offsets represent the fitting errors of the generated ephemeris, which can be seen as the errors on real-time service of the precise orbits resulting from broadcasting with the ephemeris. By analyzing the errors of the generated ephemeris broadcasting the precise orbits, the broadcast accuracy of the precise orbits can be assessed.

**Figure 2:**
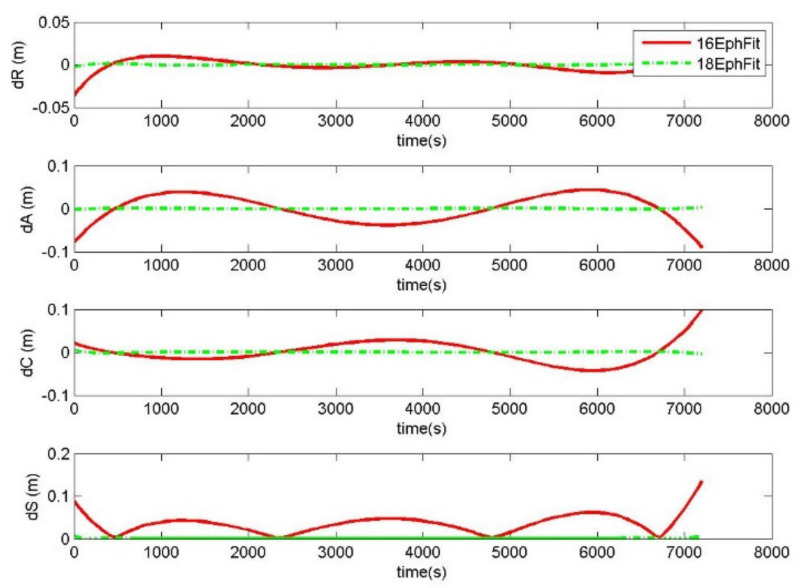
The errors of 18-parameter ephemeris and 16-parameter ephemeris fitting precise orbits (18EphFit and 16EphFit) during the 2-hour validity period for the satellite of PRN 3 starting from UTC 2017/07/05 00:00:00.

**Figure 3:**
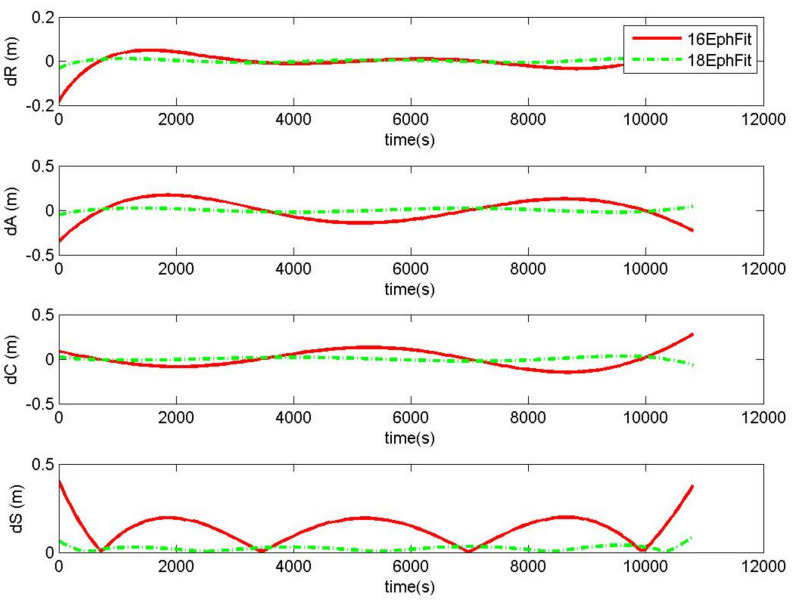
The errors of 18-parameter ephemeris and 16-parameter ephemeris fitting precise orbits (18EphFit and 16EphFit) during the 3 h validity period for the satellite of PRN 3 starting from UTC 2017/07/05 00:00:00.

In particular, the radial, along-track, and cross-track offsets *dR, dA, dC* between the calculated orbits from the generated ephemeris and the precise orbits were taken as the fitting errors. *dS* is the corresponding distance offset, where $dS=\sqrt{dR_{}^{2}+dA_{}^{2}+dC_{}^{2}}$. In the case of a single GPS satellite, Figure 2 shows the errors of 18-parameter ephemeris and 16-parameter ephemeris fitting precise orbits during the 2 h validity period. In the same manner, Figure 3 shows the fitting errors during the 3-h validity period. The RMS of fitting errors from all GPS satellites over two days was calculated to analyze the fitting accuracy. Table 1 shows the RMS of the 18-parameter ephemeris and 16-parameter ephemeris fitting errors.

The fitting accuracy of the 18-parameter ephemeris is improved by nearly eight times compared to that of the 16-parameter ephemeris. Table 1 counts the RMS of the ephemeris fitting errors from all GPS satellites over two days. The distance RMS of the 18-parameter ephemeris fitting errors within the 2 h validity period was 0.0014 m, and that of the 16-parameter ephemeris was 0.0113 m. The sequence of fitting errors from satellite 3 is shown in Figure 2. Distance offsets of 0.1 m were displayed in the 16-parameter ephemeris fitting 2 h precise orbits. This indicates that a distance error of 0.1 m may still remain after the 16-parameter ephemeris modeling precise orbits of 2 h. As the 16-parameter ephemeris lacks the change rate of semi-major axis and the rate of mean motion, more precise orbital modeling is hard to achieve. In contrast, the distance offsets of the 18-parameter ephemeris fitting 2 h precise orbits were 0.002 m. Therefore, the 18-parameter ephemeris is more suitable for broadcasting centimeter-level orbits and can be used to replace the precise orbits.

Compared to the 3 h validity period, the fitting accuracy was improved nearly six times by the 2 h validity period. In the case of the generated ephemeris fitting precise orbits during the 3 h period, Table 1 shows that the RMS of distance errors of the 18-parameter was 0.0087 m, and that of the 16-parameter ephemeris was 0.0653m. The RMS of distance errors of (0.0087, 0.0653) m were nearly magnified six times relative to the 2 h cases of (0.0014, 0.0113) m. The sequence of fitting errors during the 3 h validity period from satellite 3 is shown in Figure 3. The 16-parameter ephemeris fitting 3 h precise orbits had a 0.5 m distance offset. This demonstrates that as the fitting period increases, the fitting error increases significantly. Hence, the 2 h period is more suitable for fitting precise orbits.

The fitting stability of the 18-parameter ephemeris is distinctly better than that of the 16-parameter ephemeris. In light of the fluctuation of fitting errors, Figure 2 and Figure 3 show that the distance errors of the 16-parameter ephemeris fitting precise orbits varied obviously during the period, while those of the 18-parameter ephemeris remained stable. The 18-parameter ephemeris significantly improved the adaptability of ephemeris modeling by adding the parameters of the change rate of the semi-major axis and the rate of mean motion. Therefore, when satellites use ephemeris to broadcast precise orbits, the 18-parameter ephemeris is recommended.

### 4.2. Results of the Generated Ephemeris Predicting Precise Orbits

**Figure 4:**
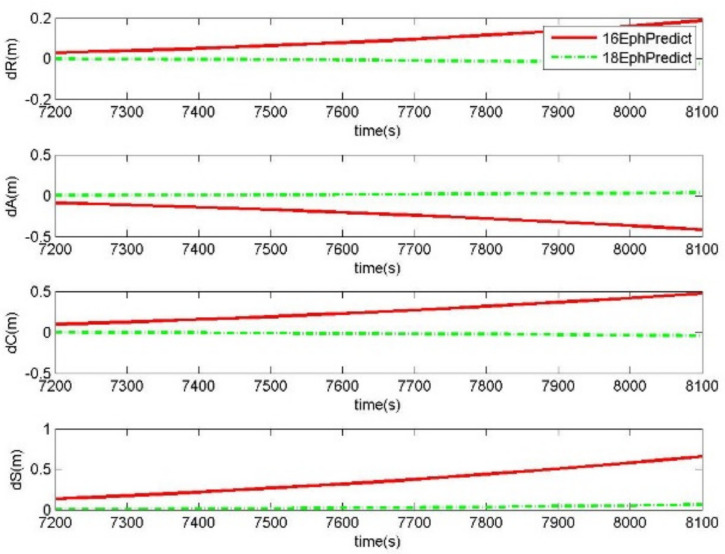
The prediction errors in 15 min after the 2 h validity period by the 18-parameter ephemeris and the 16-parameter ephemeris (18EphPredict and 16EphPredict) for satellite 3 starting from UTC 2017/07/05 00:00:00.

**Figure 5:**
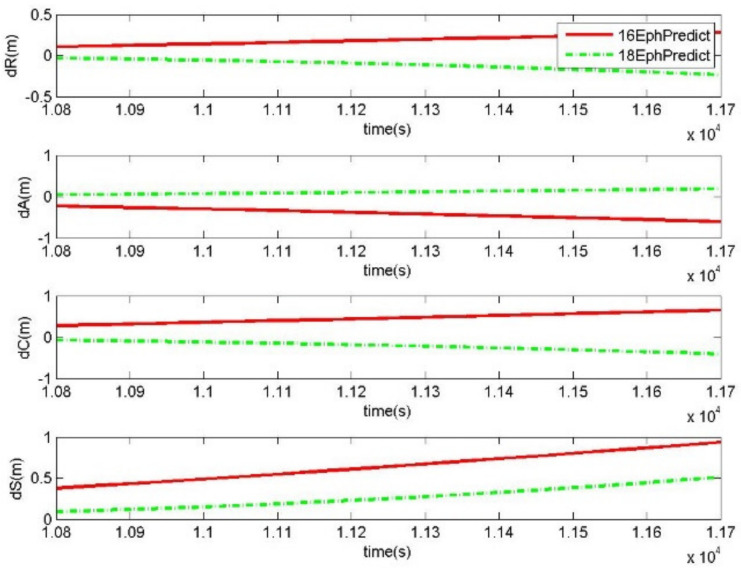
The prediction errors in 15 min after the 3 h validity period by the 18-parameter ephemeris and the 16-parameter ephemeris (18EphPredict and 16EphPredict) for satellite 3 starting from UTC 2017/07/05 00:00:00.

The prediction performances of 18-parameter ephemeris and 16-parameter ephemeris after the validity period were also tested. Taking the results from the above generation experiments, the 16-parameter ephemeris and 18-parameter ephemeris were obtained with the validity periods of 2 and 3 h, respectively. In a period of 15 min after the validity period, the satellite positions could be predicted by the 18-parameter ephemeris and the 16-parameter ephemeris. The offsets between the precise orbits and the predicted orbits by the generated ephemeris were calculated to analyze the prediction errors of the generated ephemeris. *dR*, *dA*, and *dC* are the coordinate components of the prediction errors, and *dS* is the corresponding distance error. In the case of a single satellite, Figure 4 shows the prediction errors of 18-parameter ephemeris and 16-parameter ephemeris after the 2 h validity period. In the same manner, Figure 5 exhibits the prediction errors after the 3 h validity period. RMS of prediction errors from all GPS satellites over two days were calculated to analyze the prediction accuracy. Table 2 shows the prediction accuracies of 18-parameter ephemeris and 16-parameter ephemeris.

Compared to the 16-parameter ephemeris, the prediction accuracy of the 18-parameter ephemeris had been improved nearly five times. Table 2 shows the RMS errors of the generated ephemeris predicting the orbits of 15 min after the validity periods for all GPS satellites over two days. The RMS of distance errors was 0.021 m from the generated 18-parameter ephemeris predicting 15 min orbits after the 2 h validity period, and that of the 16-parameter ephemeris was 0.1003 m. For the PRN 3 satellite, Figure 4 analyzes the sequence of prediction errors with the generated ephemeris after the 2 h validity period. In the case of the 16-parameter ephemeris predicting precise orbits, the prediction errors will rapidly increase with the prediction duration, and ranged from 0.1 m to 0.6 m within 15 min. Since the 18-parameter ephemeris has two additional parameters, which are the change rate of the semi-major axis and the rate of mean motion, the ability of orbit prediction is greatly improved. The distance errors of the 18-parameter ephemeris prediction increased to 0.05 m. Therefore, the 18-parameter ephemeris is more suitable for centimeter-level orbit prediction, which guarantees the reliable services of precise orbits when there is communication interruption and missing orbit correction streams.

Compared to the 3 h validity period, the prediction accuracy of the 2 h validity period was improved by nearly four times. Table 2 shows that the RMS of distance errors with the 18-parameter ephemeris predicting 15 min orbits after the 3 h validity period was 0.0897 m, and that of the 16-parameter ephemeris was 0.444 m, which were magnified nearly four times relative to the cases of the 2 h validity period with those of (0.021, 0.1003) m. In particular, the sequence of prediction errors from satellite 3 is shown in Figure 5. The 16-parameter ephemeris predicting 15 min orbits after the 3 h validity period had a distance offset of 1.0 m, and that of the 18-parameter ephemeris was 0.5 m. This indicates that even though the length of the fitting orbits is increased, the prediction accuracy of the generated ephemeris will not improve. Therefore, when the generated ephemeris is planned to keep the centimeter-level accuracy over the prediction orbits after the validity period, it is recommended that the fitting duration and validity period should be 2 h.

### 4.3. Results of PPP with the Generated Ephemeris during the Validity Period

During the validity period, PPP tests were carried out, and the performance of PPP with the generated ephemeris were assessed. The observation data were the dual-frequency GPS data provided by 12 globally distributed IGS stations. The observation period was two days, and the sampling interval was 30 s. As the distance error reached 0.5 m on the 16-parameter ephemeris fitting precise orbits with a 3 h validity period, the 3 h validity period is no longer suitable for real-time PPP. Therefore, only the ephemeris with the 2 h validity period was adopted to test the PPP performance. The precise clock products, the ionospheric-free phase, and pseudorange were adopted. The 16-parameter ephemeris and 18-parameter ephemeris with a 2 h validity period were generated to supply the satellite orbits.

PPP tests processed the dual-frequency GPS data from 12 IGS stations over two days. Every 2 h, PPP restarted initialization. PPP results were obtained by taking precise clock products and the generated ephemeris within the 2 h validity period. Results of PPP with the generated ephemeris were compared with the known coordinates, and the absolute positioning errors in the north, east, and up components *dN*, *dE*, and *dU* were obtained. In the epoch-by-epoch approach, the relative positioning offsets are obtained between the results of PPP with the precise orbits and the results of PPP with the generated ephemeris. The relative positioning offsets represent the difference in PPP results because of the generated ephemeris broadcasting the precise orbits. The RMS of the positioning offsets or positioning errors can be counted. Table 3 shows the RMS of the positioning offsets.

The absolute positioning errors between the PPP results with the generated ephemeris and the known coordinates from Table 3, show that the RMS of the distance error of PPP with the 18-parameter ephemeris broadcasting precise orbits could achieve 0.0539 m and that of the 16-parameter ephemeris reached 0.1015 m. Therefore, the generated ephemeris broadcasts precise orbits that can achieve centimeter-level PPP accuracy.

The epoch-by-epoch relative offsets between the results of PPP with the precise orbits and the results of PPP with the generated ephemeris from Table 3, show that the RMS of the distance offsets of PPP with the 18-parameter ephemeris was about 0.009 m, and that of the 16-parameter ephemeris was about 0.077 m. The RMS of the positioning offsets indicate that the results of PPP with the 18-parameter ephemeris had no significant difference to that of precise orbits. It was validated that the generated ephemeris broadcasting precise orbits was applicable to real-time PPP. Therefore, the difference in PPP due to the generated 18-parameter ephemeris instead of precise orbits is 1 cm. Substantially, for real-time PPP applications of 5 cm accuracy, the generated ephemeris is feasible to utilize to broadcast precise orbits, and the PPP results with the 18-parameter ephemeris were more consistent with those of the precise orbits.

### 4.4. Results of PPP with the Generated Ephemeris after the Validity Period

After the validity period, PPP tests were carried out, and the performance of PPP with the predicted orbits from the generated ephemeris was assessed. The dual-frequency GPS data from 12 stations over two days were processed by PPP. The starting time of PPP was set as 2N o’clock (N = 0, 1, …, 11), and each processing period was 2 h and 15 min, where the generated ephemeris supplies the calculated orbits within a 2 h validity period and the predicted orbits of 15 min after the 2 h validity period. Furthermore, the precise clock products and the ionospheric-free phase and pseudorange were adopted by PPP.

Results of PPP with the predicted orbits from the generated ephemeris were compared with the known coordinates, and the absolute positioning errors in the north, east, and up components *dN*, *dE*, and *dU* were obtained. In the epoch-by-epoch approach, the relative positioning offsets were obtained between the results of PPP with the precise orbits and results of PPP with the predicted orbits of 15 min after the 2 h validity period from the generated ephemeris. The RMS of the positioning errors and positioning offsets were counted and are exhibited in Table 4.

After 2 h, the PPP results have completely converged, and the difference between the PPP results and the known positions is small and stable, and the RMS of positioning errors after 2 h is smaller than those before 2 h. Table 3 shows that the RMS of the positioning errors from the predicted orbits of 15 min after the 2 h validity period by the 18-parameter ephemeris was about 0.0251 m. This implies that the predicted orbits of 15 min by the generated ephemeris can achieve centimeter-level PPP accuracy.

In Table 3, RMS of the positioning offsets between the results of PPP with precise orbits and the results of PPP with the predicted orbits of 15 min after the 2 h validity period by the 16-parameter ephemeris was about 0.0287 m. This indicates that the PPP results with the predicted orbits by the 16-parameter ephemeris had a significant difference from those of precise orbits. However, RMS of the positioning offsets between the results of PPP with precise orbits and the results of PPP with the predicted orbits of 15 min after the 2 h validity period by the 18-parameter ephemeris was about 0.0057 m. Therefore, the results of PPP with the predicted orbits by the 18-parameter ephemeris had a sub-centimeter difference from those of precise orbits. This confirms that the predicted orbits from the 18-parameter ephemeris can be used to substitute the precise orbits in 15 min after the 2 h validity period. Therefore, the 18-parameter ephemeris has a better predicting ability, which can effectively guarantee real-time PPP in the case of missing orbit corrections up to 15 min in RTCM mode.

## 5. Conclusions

Precise point positioning (PPP) has been developed into real-time services. For real-time PPP with a receiver or smartphone, the convenient, continuous, and reliable acquisition of precise orbits is critical to ensure stable PPP services. RTCM provides a mode of broadcasting orbit corrections to supply the real-time services of precise orbits, but this mode finds it difficult to overcome the continuity and reliability degradation caused by the loss of orbit corrections due to the occasional communication failure, and a large volume of data needs to be broadcast. In order to overcome the above problems of RTCM mode, the navigation ephemeris was generated as an alternative and improved mode to broadcast the precise orbits with centimeter-level precision in real-time. Although navigation ephemeris has been adopted for broadcasting satellite orbits with 1 m precision on navigation signals and serves as standard point positioning with meter-level precision and precise relative positioning. However, the potential of navigation ephemeris can be further studied and extended to real-time PPP.

With a different background and purpose from the existing navigation ephemeris investigations, this study proposed a service mode in which the ephemeris parameters were generated to broadcast the ultra-rapid precise orbits with centimeter-level precision, and directly apply to the centimeter-level real-time PPP for the receiver, smartphone, and others. For the first time in GPS, this paper provides the difference in PPP results between the precise orbits and the calculated orbits with the generated ephemeris parameters, and the deviations resulted from the mode of broadcasting precise orbits with the generated ephemeris were carefully evaluated and analyzed in terms of orbits errors and positioning offsets. Consequently, some helpful findings are supplied to real-time PPP. (1) Within the validity period of 2 h, the RMS of relative distance offsets between the results of PPP with the precise orbits and the results of PPP with the calculated orbits by the 18-parameter ephemeris was 1 cm. This implies that the mode of broadcasting precise orbits with 18-parameter ephemeris is completely suitable and feasible for real-time PPP. (2) Within 15 min after the validity period of 2 h, the RMS of relative distance offsets between the results of PPP with the precise orbits and the results of PPP with the predicted orbits by the 18-parameter ephemeris was 0.6 cm. This confirms that the predicted orbits by the 18-parameter ephemeris within 15 min after the validity period can replace the precise orbits. (3) There was no significant difference between the precise orbits and the calculated orbits with the 18-parameter ephemeris during the 2 h validity period. The RMS of distance errors was 1.4 mm in the 18-parameter ephemeris fitting precise orbits during the 2 h validity period. (4) The 18-parameter ephemeris can provide centimeter-level prediction orbits of 15 min after the 2 h validity period. The RMS of distance error was 2.1 cm in the 18-parameter ephemeris predicting orbits of 15 min after the 2 h validity period.

Compared to the classic RTCM mode of broadcasting precise orbits with orbit corrections, the service mode of 18-parameter ephemeris broadcasting precise orbits achieves following advantages. (1) The amount of broadcast data was reduced by 20 times, since only 18 parameters are needed to broadcast within the 2 h validity period. (2) The validity period was expanded by 120 times and increased to 2 h. Thereby, the interruption of real-time services of precise orbits caused by the transmission failure of RTCM correction data stream during the validity period is avoided, and the continuity and reliability of real-time services of precision orbits are improved. (3) Within 15 min after the validity period, the predicted orbits are provided with a precision of 5 cm. Thus, the interruption of the real-time services of precise orbits induced by the transmission failure within 15 min after the validity period are overcome, and the reliability of the real-time services of the precise orbits is ensured. (4) Without additional correction operation on satellite position, the receiver and smartphone can easily obtain the precise orbits with the calculation algorithm the same as the navigation ephemeris, which improves the convenience of real-time services of precise orbits. In summary, the convenience, continuity, and reliability are improved in the real-time services of precise orbits broadcast by the 18-parameter ephemeris.

Finally, the service mode of the 18-parameter ephemeris transmitting precise orbits was validated from two fields. The first validation from the orbital field was the fitting and predicting tests on all GPS satellites over two days with the generated ephemeris broadcasting precise orbits. The second validation from the positioning field was the PPP tests with the generated ephemeris. In the orbital validation, the feasibility of 18-parameter ephemeris broadcasting precise orbits was verified, which was validated by the fitting accuracy during the ephemeris validity period, the prediction accuracy after the validity period, and the suggested length of validity period. In the positioning validation, the availability of PPP with the generated ephemeris was verified, which was validated by the positioning offsets between PPP with the precise orbits and PPP with the calculated orbits from the generated ephemeris during the 2 h validity period; the positioning offsets between PPP with the precise orbits and PPP with the predicted orbits of 15 min after the 2 h validity period from the generated ephemeris; and the positioning errors between the known positions and the results of PPP with the generated ephemeris. However, this study was limited to MEO satellites and PPP tests of GPS, which has certain representativeness in Global Navigation Satellite System (GNSS) applications. In the future, it is recommended to traverse the massive orbits data of all GPS satellites and conduct PPP tests from globally covered stations.

## Figures and Tables

**Table 1:** Root mean square (RMS) of the 18-parameter ephemeris and 16-parameter ephemeris fitting errors from all Global Position System (GPS) satellites over two days.

Ephemeris Type	Validity Period (hour)	RMS (*dR*)	RMS (*dA*)	RMS (*dC*)	RMS (*dS*)
16-parameter ephemeris	2	0.0033	0.0076	0.0072	0.0113
18-parameter ephemeris	2	0.0005	0.0009	0.0009	0.0014
16-parameter ephemeris	3	0.0225	0.0426	0.042	0.0653
18-parameter ephemeris	3	0.0034	0.0052	0.0057	0.0087

**Table 2:** The RMS of prediction errors in 15 min by the 18-parameter ephemeris and 16-parameter ephemeris from all GPS satellites over two days.

Ephemeris Type	Validity Period (hour)	RMS (*dR*)	RMS (*dA*)	RMS (*dC*)	RMS (*dS*)
16-parameter ephemeris	2	0.0416	0.0626	0.0559	0.1003
18-parameter ephemeris	2	0.0088	0.0099	0.0139	0.021
16-parameter ephemeris	3	0.2014	0.2483	0.2625	0.444
18-parameter ephemeris	3	0.044	0.0425	0.055	0.0897

**Table 3:** Statistical results of the absolute positioning errors and relative positioning offsets with the generated ephemeris within the 2-h validity period.

RMS (m)	RMS (*dN*)	RMS (*dE*)	RMS (*dU*)	RMS (*dS*)
RMS of absolute positioning errors between the known positions and the results of PPP with the calculated orbits from the generated ephemeris				
Results of PPP with 16-parameter ephemeris minus the known positions	0.0297	0.0571	0.0703	0.1015
Results of PPP with 18-parameter ephemeris minus the known positions	0.0129	0.0424	0.0278	0.0539
RMS of relative positioning offsets between the results of PPP with the precise orbits and the results of PPP with the calculated orbits from the generated ephemeris				
Results of PPP with 16-parameter ephemeris minus those of precise orbits	0.0271	0.0237	0.0633	0.0771
Results of PPP with 18-parameter ephemeris minus those of precise orbits	0.0035	0.0033	0.008	0.0098

**Table 4:** Statistical results of the absolute positioning errors and relative positioning offsets with the generated ephemeris predicting of 15 min orbits after the 2 h validity period.

	RMS (*dN*)	RMS (*dE*)	RMS (*dU*)	RMS (*dS*)
RMS of absolute positioning errors between the known positions and the results of PPP with the predicted orbits from the generated ephemeris				
Results of PPP with 16-parameter ephemeris minus the known positions	0.0142	0.0264	0.028	0.0429
Results of PPP with 18-parameter ephemeris minus the known positions	0.0072	0.0155	0.0159	0.0251
RMS of relative positioning offsets between the results of PPP with the precise orbits and the results of PPP with the predicted orbits from the generated ephemeris				
Results of PPP with 16-parameter ephemeris minus those of precise orbits	0.0097	0.0136	0.0204	0.0287
Results of PPP with 18-parameter ephemeris minus those of precise orbits	0.0013	0.0026	0.0044	0.0057

## Appendix A

The partial derivatives of the satellite coordinate component $R_{i}$ with respect to the corresponding ephemeris parameters are deduced as:

(A1) $$\frac{\partial R_{i}}{\partial \Delta A}=\frac{\partial R_{i}}{\partial r}.\frac{\partial r}{\partial A}.\frac{\partial A}{\partial \Delta A}+\frac{\partial R_{i}}{\partial u}.\frac{\partial u}{\partial A}.\frac{\partial A}{\partial \Delta A}+\frac{\partial R_{i}}{\partial i}.\frac{\partial i}{\partial A}.\frac{\partial A}{\partial \Delta A}$$

(A2) $$\frac{\partial R_{i}}{\partial \dot{A}}=\frac{\partial R_{i}}{\partial r}.\frac{\partial r}{\partial A}.\frac{\partial A}{\partial \dot{A}}+\frac{\partial R_{i}}{\partial u}.\frac{\partial u}{\partial A}.\frac{\partial A}{\partial \dot{A}}+\frac{\partial R_{i}}{\partial i}.\frac{\partial i}{\partial A}.\frac{\partial A}{\partial \dot{A}}$$

(A3) $$\frac{\partial R_{i}}{\partial e}=\frac{\partial R_{i}}{\partial r}.\frac{\partial r}{\partial e}+\frac{\partial R_{i}}{\partial u}.\frac{\partial u}{\partial e}+\frac{\partial R_{i}}{\partial i}.\frac{\partial i}{\partial e}$$

(A4) $$\frac{\partial R_{i}}{\partial i_{0}}=\frac{\partial R_{i}}{\partial i}.\frac{\partial i}{\partial i_{0}}$$

(A5) $$\frac{\partial R_{i}}{\partial \Omega_{0}}=\frac{\partial R_{i}}{\partial \Omega }.\frac{\partial \Omega }{\partial \Omega_{0}}$$

(A6) $$\frac{\partial R_{i}}{\partial w}=\frac{\partial R_{i}}{\partial r}.\frac{\partial r}{\partial w}+\frac{\partial R_{i}}{\partial u}.\frac{\partial u}{\partial w}+\frac{\partial R_{i}}{\partial i}.\frac{\partial i}{\partial w}$$

(A7) $$\frac{\partial R_{i}}{\partial M_{0}}=\frac{\partial R_{i}}{\partial r}.\frac{\partial r}{\partial M_{0}}+\frac{\partial R_{i}}{\partial u}.\frac{\partial u}{\partial M_{0}}+\frac{\partial R_{i}}{\partial i}.\frac{\partial i}{\partial M_{0}}$$

(A8) $$\frac{\partial R_{i}}{\partial (\Delta n_{0}^{})}=\frac{\partial R_{i}}{\partial r}.\frac{\partial r}{\partial (\Delta n)}.\frac{\partial \Delta n}{\partial (\Delta n_{0}^{})}+\frac{\partial R_{i}}{\partial u}.\frac{\partial u}{\partial (\Delta n)}.\frac{\partial \Delta n}{\partial (\Delta n_{0}^{})}+\frac{\partial R_{i}}{\partial i}.\frac{\partial i}{\partial (\Delta n)}.\frac{\partial \Delta n}{\partial (\Delta n_{0}^{})}$$

(A9) $$\frac{\partial R_{i}}{\partial \dot{i}}=\frac{\partial R_{i}}{\partial i}.\frac{\partial i}{\partial \dot{i}}$$

(A10) $$\frac{\partial R_{i}}{\partial c_{us}}=\frac{\partial R_{i}}{\partial u}.\frac{\partial u}{\partial c_{us}}$$

(A11) $$\frac{\partial R_{i}}{\partial c_{uc}}=\frac{\partial R_{i}}{\partial u}.\frac{\partial u}{\partial c_{uc}}$$

(A12) $$\frac{\partial R_{i}}{\partial c_{is}}=\frac{\partial R_{i}}{\partial i}.\frac{\partial i}{\partial c_{is}}$$

(A13) $$\frac{\partial R_{i}}{\partial c_{ic}}=\frac{\partial R_{i}}{\partial i}.\frac{\partial i}{\partial c_{ic}}$$

(A14) $$\frac{\partial R_{i}}{\partial c_{rs}}=\frac{\partial R_{i}}{\partial r}.\frac{\partial r}{\partial c_{rs}}$$

(A15) $$\frac{\partial R_{i}}{\partial c_{rc}}=\frac{\partial R_{i}}{\partial r}.\frac{\partial r}{\partial c_{rc}}$$

(A16) $$\frac{\partial R_{i}}{\partial (\Delta n_{0}^{})}=\frac{\partial R_{i}}{\partial r}.\frac{\partial r}{\partial (\Delta n)}.\frac{\partial \Delta n}{\partial (\Delta n_{0}^{})}+\frac{\partial R_{i}}{\partial u}.\frac{\partial u}{\partial (\Delta n)}.\frac{\partial \Delta n}{\partial (\Delta n_{0}^{})}+\frac{\partial R_{i}}{\partial i}.\frac{\partial i}{\partial (\Delta n)}.\frac{\partial \Delta n}{\partial (\Delta n_{0}^{})}$$

(A17) $$\frac{\partial R_{i}}{\partial (\Delta {\dot{n}}_{0}^{})}=\frac{\partial R_{i}}{\partial r}.\frac{\partial r}{\partial (\Delta n)}.\frac{\partial \Delta n}{\partial (\Delta {\dot{n}}_{0}^{})}+\frac{\partial R_{i}}{\partial u}.\frac{\partial u}{\partial (\Delta n)}.\frac{\partial \Delta n}{\partial (\Delta {\dot{n}}_{0}^{})}+\frac{\partial R_{i}}{\partial i}.\frac{\partial i}{\partial (\Delta n)}.\frac{\partial \Delta n}{\partial (\Delta {\dot{n}}_{0}^{})}$$

(A18) $$\frac{\partial R_{i}}{\partial \Delta \dot{\Omega }}=\frac{\partial R_{i}}{\partial \Omega }.\frac{\partial \Omega }{\partial \Delta \dot{\Omega }}$$

Among the above equations, $R_{i}$ (*i* = 1, 2, 3) is the coordinate component of satellite position in earth-centered, earth-fixed coordinate (ECEF). *r* is the distance between the satellite and earth center.
